# Clinicopathological features and prognosis of ciliated muconodular papillary tumor

**DOI:** 10.1186/s13019-019-0962-3

**Published:** 2019-07-24

**Authors:** Kang Shao, Yalong Wang, Qi Xue, Juwei Mu, Yushun Gao, Yonggang Wang, Bingzhi Wang, Lina Zhou, Shugeng Gao

**Affiliations:** 1 Department of Thoracic Surgery, National Cancer Center/ National Clinical Research Center for Cancer/ Cancer Hospital, Chinese Academy of Medical Sciences and Peking Union Medical College, Beijing, 100021 People’s Republic of China; 2 Department of Pathology, National Cancer Center/ National Clinical Research Center for Cancer/ Cancer Hospital, Chinese Academy of Medical Sciences and Peking Union Medical College, Beijing, 100021 People’s Republic of China; 3 Department of Diagnostic Radiology, National Cancer Center/ National Clinical Research Center for Cancer/ Cancer Hospital, Chinese Academy of Medical Sciences and Peking Union Medical College, Beijing, 100021 People’s Republic of China

**Keywords:** Ciliated muconodular papillary tumor, Lung, Prognosis

## Abstract

**Backgrounds:**

The pulmonary ciliated muconodular papillary tumor (CMPT) is a very rare tumor with only several case reports in published literatures, and its clinicopathological features, standard treatment methods and prognosis has not been well defined.

**Methods:**

Two cases of CMPT diagnosed and treated in our hospital and 39 cases reported in the published literature were analyzed retrospectively.

**Results:**

The cohort of 41 CMPT patients comprised of 20 males and 21 females, aged 9–84 years. The diameter of the primary tumor was 0.3–4.5 cm. Most of these lesions were subsolid nodules, as observed on computed tomography and easily misdiagnosed as early lung adenocarcinoma. Tumors of 26 patients were stained by immunohistochemistry method, which revealed that CK7, CEA, and TTF-1 were positive and CK20 was negative in most patients. The results of gene alternation demonstrated mutations in *EGFR*, *KRAS*, and *BRAF* and *ALK* rearrangements in CMPT. All the patients underwent surgical treatment and did not receive postoperative adjuvant therapy. The follow-up duration was 0–120 months, and no case of tumor recurrence was found until the final follow-up.

**Conclusions:**

The incidence of CMPT was low and rate of image misdiagnosis high. Immunohistochemistry is helpful for accurate diagnosis of CMPT. Sub-lobectomy may be proper and adjuvant treatment should be avoided since the disease is now prone to benign lesions. Furthermore, since the biological behavior of this tumor is not yet fully elucidated, additional case data are essential for accurate conclusions.

## Background

Ishikawa [1], for the first time, reported a rare case of lung tumor in 2002. The tumor was 1.5 cm and located in the periphery lung. Microscopically, it exhibited papillary structure and abundant ciliated columnar cells, goblet cells, and basal cells; the alveolar cavity around the primary lesion was filled with mucus lakes. This tumor proliferated slowly and lacked nuclear atypia. According to the morphological characteristics and clinical manifestations, it was termed as the ciliated muconodular papillary tumor [2]. The incidence of CMPT is very low; only a few cases have been reported that primarily include Asian population. The nomenclature of CMPT has not yet been classified into World Health Organization lung tumor classification. Therefore, there is a lack of knowledge about the disease among clinical and pathological doctors. The reasonable diagnosis and treatment modes are still under exploration [3]. Herein, we retrospectively summarized the clinicopathological data of 41 CMPT cases to provide diagnosis and treatment reference for clinicians.

## Patients and methods

### Patients

Two cases of CMPT were treated in our hospital during September 2017 and March, 2018. The tumor specimens of both patients were obtained with informed consent, and underwent immunohistochemistry staining, and the pathological sections were reviewed by two chief pathologists. Both cases were eventually diagnosed as CMPT. We performed a comprehensive literature search for published articles using the WANGFANG DATA in Chinese, PubMed and Clinicalkey in English. A total of 13 articles (1 in WANFANG DATA and 12 in PubMed and Clinicalkey) were eventually included, and the data from 39 cases, reported in the literature, were analyzed statistically. This study was approved by the medical ethics committee of our hospital.

### Clinical information and follow-up

Clinical data of two patients in our center were obtained from the medical record database center, while the data of other 39 patients were collected according to the description of relevant literatures. Clinical data included nationality, age, sex, smoking history, location of the lesion, diameter of the tumor, follow-up time and survival. Two patients in our hospital were followed up on the telephone until May 26, 2018. The follow-up duration of the other 39 patients was described in the relevant literature.

## Results

### Clinical features of 41 CMPT patients

**Fig. 1:**
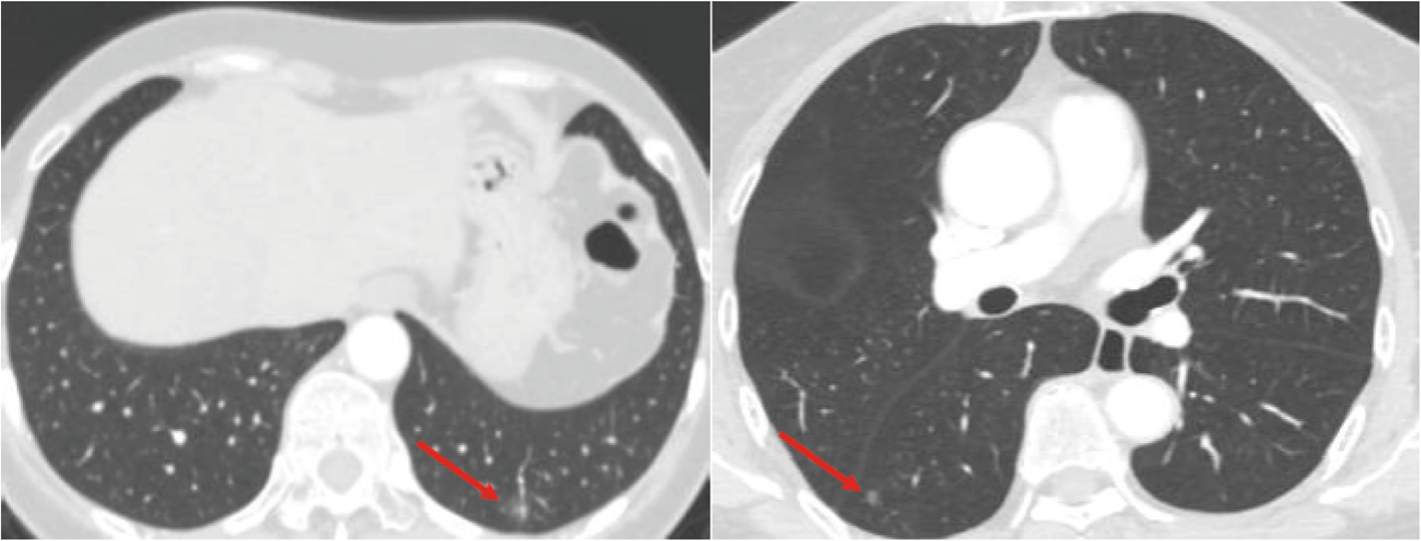
CT images of 2 CMPT patients treated at our hospital (left side: case 1, right side: case 2)

The clinical data of 2 patients in our hospital and 39 cases reported in the literature are summarized in Tables 1 and 2, respectively [1–14]. Among the previously reported 39 CMPT patients, 30 were from Japan, 1 from Singapore, 4 from America, and 4 from China. The cohort comprised of 20 males and 19 females with a median age of 66 (9–84) years. The median tumor diameter was 1.0 (0.3–4.5) cm. The tumors were located in the inferior lobe in 25 cases, upper lobe in 7, and middle lobe in 1, while the location was unknown in 6 cases. Most of the lesions were detected during the regular physical examination or opportunistic screening, and thus patients had no complaints. In the current group, the first patient had a family history of malignant tumor (father with liver cancer, mother with ureteral cancer and gastric cancer, brother with colon cancer) and exhibited 2 subsolid ground-glass lung nodules (one in the left inferior lobe and one in the right inferior lobe with a diameter of 1.5 cm) and several small pure ground-glass lung nodules by chest computed tomography (CT) scan in a routine examination; the second patient was found to have two nodules (one was confirmed to be lung adenocarcinoma, and the other was CMPT) in the lung by chest CT scan during regular examination after an operation of thyroid carcinoma 1 year ago. The CMPT mostly appears as subsolid nodules with a distinct or irregular shape in the CT image and can be easily diagnosed as lung adenocarcinoma. The 2 cases in our group presented small nodules, of which, one composed of ground glass components (Fig. 1).

**Table 1:** Clinicopathological features of 2 CMPT patients at our hospital

No.	Sex	Age (years)	Complaint	location	Diameter (cm)	Imaging features	Surgery	Immunohistochemistry
1	female	58	examination	left inferior lobe	0.8	ground glass nodule	wedge resection	AE1/AE3 (3+), CEA (+), TTF-1 (3+), CK7 (3+), C-MET (2+), MUC5AC (+), P63 (basal cells 3+), P53 (partially +), BRAF-V600E (−), EGFR (2+), HER2 (−), ROS1 (−), Ki-67 (10%)
2	female	66	examination	right inferior lobe	0.6	nodule	wedge resection	TTF-1 (3+), CK7 (3+), P63 (3+), CK5/6 (3+), MUC5AC (1+), CK20 (−), Ki-67 (2%)

**Table 2:** Clinicopathological features of 39 CMPT patients reported in the literature

Features	Cases
Publication time	
2002–2014	6
2015	10
2016	15
2017	8
Nationality	
Japan	30
Singapore	1
USA	4
China	4
Age (years)	
19	1
50–60	7
60–70	14
70–80	8
≥80	5
unknown	4
Sex	
male	20
female	19
Smoking history	
yes	13
No	16
unknown	10
Location of the lesion	
right upper lobe	5
right middle lobe	1
right inferior lobe	15
left upper lobe	2
left inferior lobe	10
unknown	6
Diameter (cm)	
< 1.0	16
1.0–2.0	17
≥2.0	2
unknown	4
Median follow-up time (months)	
25 (0–120)	39
Survival status	
recurrence	0
without recurrence	39

### Pathological features of 2 patients treated at our hospital

#### Case 1

**Fig. 2:**
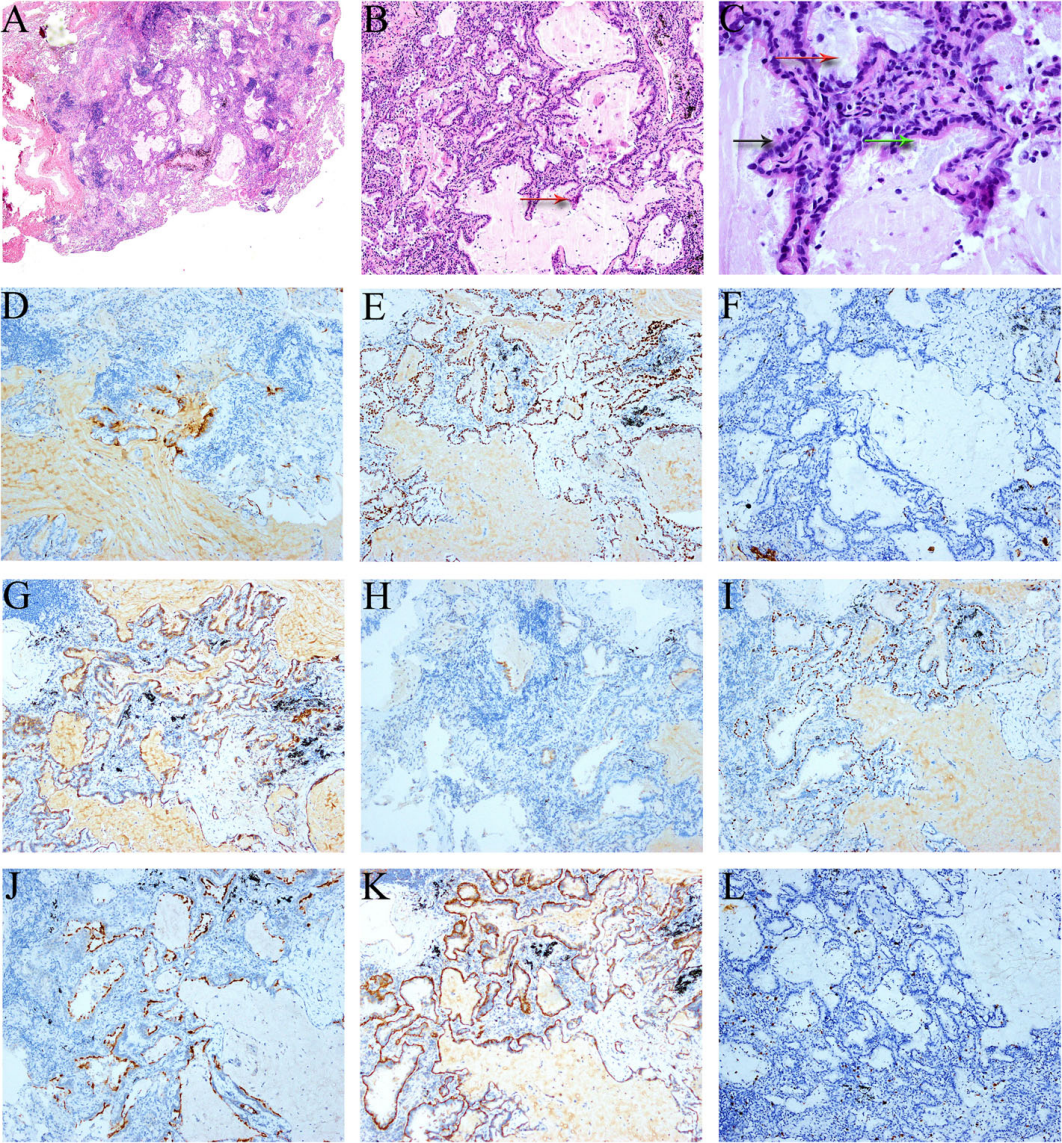
Histopathological and immunohistochemistry findings in case 1 (**a** HE× 20; **b** HE × 100, red arrow: papillary structure; **c** HE × 400, red arrow: mucous pool, black arrow: goblet cell, green arrow: ciliated cells; **d** CEA (+); **e** TTF-1 (3+); **f** Napsin A (3+); **g** C-MET (2+); **h** MUC5AC (+); **i** P63 (basal cells 3+), **j** BRAF-V600E (−); **k** EGFR (2+); **l** Ki-67 (+,10%))

Grossly, the size of the wedge-shaped lung was 4.0 × 3.0 × 2.0 cm^3^. A grey, solid, and hard nodule (0.8 cm) was observed in the section of the lung. The tumor had a clear boundary and was not involved in visceral pleura. At high magnification, the tumor was not enveloped, and a relatively clear boundary between the tumor and the surrounding tissue was evident. No obvious necrosis was detected in the tumor. At low magnification, the tumor cells arrayed irregularly along the alveolar wall and were tubular. The alveolar septum was broadened, and papillary protuberances were visible. The tumor cells lacked atypia and showed intensely stained nuclei rarely comprising of mitotic structures. Also, the tumor comprised of different cell types including mucous cells, ciliated cells, and basal cells. Immunohistochemistry analysis showed AE1/AE3 (3+), CEA (+), TTF-1 (3+), CK7 (3+), C-MET (2+), MUC5AC (+), P63 (basal cells 3+), P53 (partially +), BRAF-V600E (−), EGFR (2+), HER2 (−), ROS1 (−), Ki-67 (+, 10%) (Fig. 2). The pathological analysis of mediastinal lymph nodes sampling did not find any tumor metastasis. Genes of *EGFR*, *KRAS* and *BRAF* were sequenced and the result demonstrated a gene mutation of *KRAS-G12D* and *KRAS-G12A* in case 1*.*

#### Case 2

**Fig. 3:**
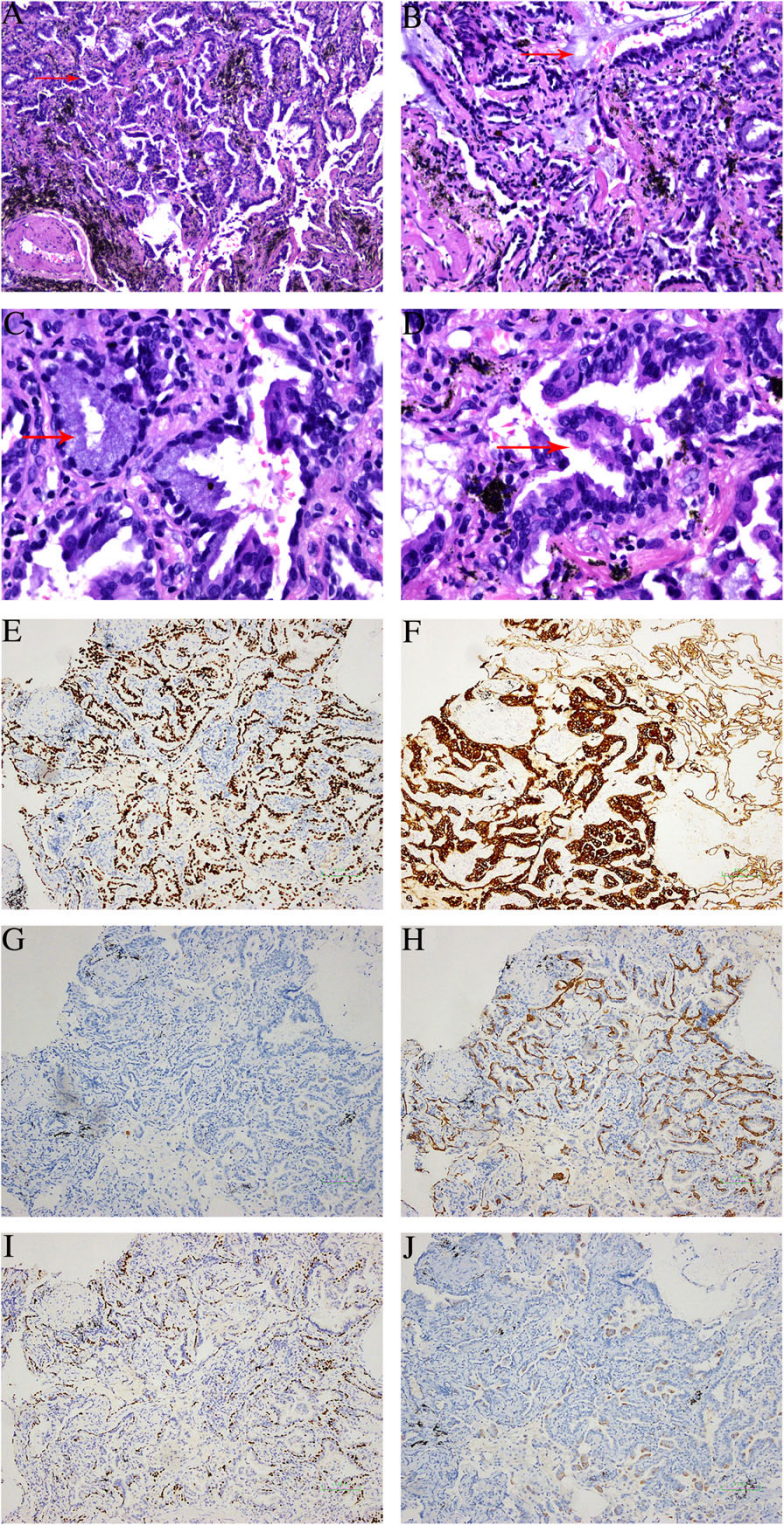
Histopathological and immunohistochemistry findings in case 2 (**a** HE × 100, red arrow: papillary structure; **b** HE × 200, red arrow: mucous pool; **c** HE × 400, red arrow: goblet cell; **d** HE × 400, red arrow: ciliated cells; **e** TTF-1(3+); **f** CK7(3+); **g** CK20(−); **h** CK5/6 (basal cells 3+), **i** P63 (basal cells 3+), **j** MUC5AC (1+))

Grossly, the size of the wedge-shaped lung was 1.5 × 2.0 × 3.0 cm^3^. A grey solid and hard nodule (0.4–0.6 cm) was observed in the section of the lung. The tumor had a clear boundary and was not involved in visceral pleura. At high magnification, the tumor was not enveloped and a relatively clear boundary was detected between the tumor and the surrounding tissue. No obvious necrosis was seen in the tumor. At low magnification, the tumor arrayed tubular structures, the mucus was visible in the lumen, and micropapillary tumor cells were found in the mucus. In addition, the tumor cells lacked atypia and showed intensely stained nuclei with rare mitosis. The tumor comprised of various cell types including mucous cells, ciliated cells, and basal cells. Immunohistochemistry showed TTF-1 (3+), CK7 (3+), P63 (basal cells 3+), CK5/6 (basal cells 3+), MUC5AC (1+), CK20 (−), and Ki-67 (2%) (Fig. 3). The pathological analysis of mediastinal lymph nodes dissection did not find any tumor metastasis. We sequenced for gene mutation in *BRAF, EGFR, KRAS, MET, ERBB2, ALK, ATK1, CTNNB1, DDR2, ERBB4, FBXW7, FGFR1, FGFR2, FGFR3, MAP2K1, NOTCH1, NRAS, PIK3CA, PTEN, SMAD4, STK11, TP5*2 and fusion gene of *ALK, RET, ROS1* and *NTR*K in this case. The result suggested that the tumor had a *BRAF-V600E (COSM476)* mutation.

### Immunohistochemistry results

Tumors of 26 patients were stained by immunohistochemistry method, the results showed that CK7 was expressed in all cases, while CEA and TTF-1 were expressed in most cases. P63, CK5/6, and P40 were expressed in the basal cells, while MUC5AC was expressed in ciliated columnar cells. The Ki-67 index of most CMPT was low and usually < 3%.

### Results of genetic alterations

**Fig. 4:**
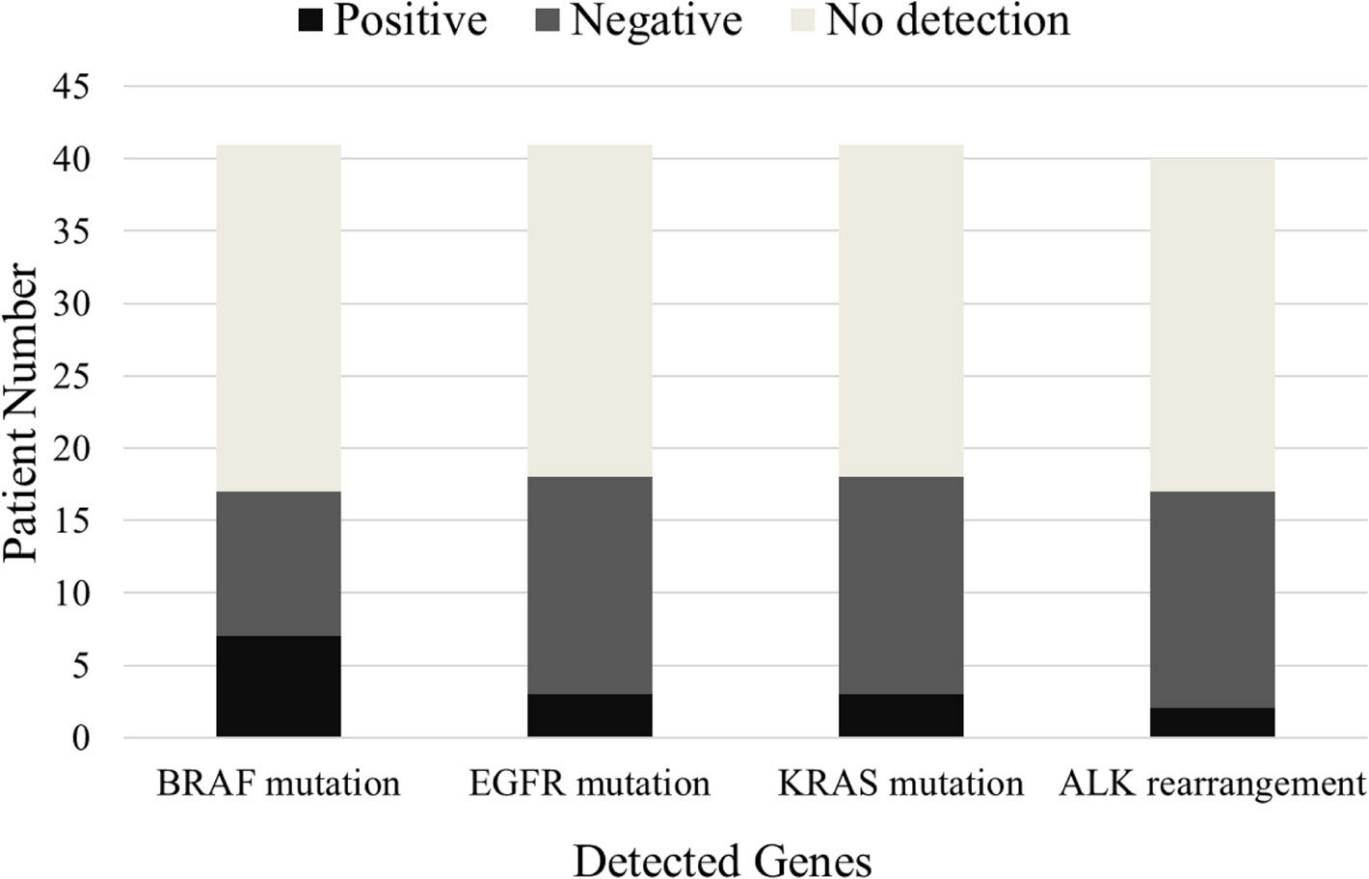
Sequencing of driving mutation in 41 CMPT patients

Of all the 39 patients, 17 underwent different panels of gene alternation sequencing. The status of driving mutation of previous 39 patients and 2 patients treated at our department were as follows: *BRAF-V600E (COSM476)* mutation was detected in 6 cases; *BRAF-G606R (COSM27640)* was positive in 1 case and negative in 10; *EGFR* exon 19 deletion was positive in 3 cases and *EGFR* mutation was negative in 15; *KRAS-G12D* was positive in 1 case, *KRAS-G12D* and *KRAS-G12A* was positive at the same time in 1 case, and *KRAS G12C* was positive in another case while *KRAS* was negative in 15; *ALK* rearrangement was positive in 2 cases and negative in 15 cases (Fig. 4). Other gene mutations were as follows: *IDH1* in 1 case, *CTNNB* in 1 case, *PTPN11* in 2 cases, *TP53* in 1 case, and *AKT1* in 2 cases.

### Treatment and prognosis

All patients underwent surgery. Nine patients underwent lobectomy, 30 patients underwent sub-lobectomy of the tumor, and the operation methods of 2 cases were not mentioned. The median follow-up time was 25 (0–120) months and no recurrence was observed.

## Discussion

CMPT was first reported in 2002 and termed as an independent tumor. Since then, a few CMPT cases have been reported. A malignant pulmonary tumor containing cilium is yet a rarity. Hitherto, only a few studies have reported that the well-differentiated papillary lung adenocarcinoma contains cilia [15–17]. Although lung tumors containing ciliated cells are usually benign, CMPT still shows some low-grade malignant tumor features such as destroyed alveolar structures and central fibrosis, proliferation along the alveolar walls and skip lesions similar to adenocarcinoma in situ, no encapsulation, and positive carcinoembryonic antigen staining [1]. Due to the low incidence of CMPT and limited reports, its clinicopathological features and malignant potential are yet to be elucidated.

CMPT is often observed in middle-aged and elderly individuals; however, a case of a 19-year-old girl was reported in the literature [12]. CMPT can occur in both men and women. Furthermore, no clear correlation was established between smoking and CMPT to date. A majority of the CMPT patients were reported as Asians; nevertheless, some cases were also reported in Western countries [13]. CMPT patients are often asymptomatic, and the lesions are mostly found through physical examination or opportunistic screening. The imaging features of CMPT are characterized as small peripheral pulmonary nodules with regular or irregular boundaries, and the lesions of some cases may contain a ground glass component or a cavity-like structure [5]. The diameter of the lesions varied, and the median diameter was 1 cm. CMPT can hardly be diagnosed by single imaging; such patients were usually diagnosed with early lung adenocarcinoma and recommended surgical treatment.

Macroscopically, CMPT is a tan-white nodule located in the peripheral lung. The section of the CMPT tumor is soft and the lesion filled with mucus [18]. Microscopically, CMPT consisted of ciliated columnar cells, goblet cells, and basal cells. Tumors may show different growth patterns, such as acinar, papillary, micropapillary pattern, or even proliferation along the alveolar walls and skip lesions similar to lung adenocarcinoma. The immunohistochemistry staining showed that TTF-1, CK7, and CEA were mostly positive and CK20 was almost negative. P63, P40, and CK5/6 were positive in basal cells, while MUC5AC was positive in ciliated cells. Some studies used in situ hybridization to identify the CMPT with human papillomavirus infection; the results revealed 10 patients negative to the detection [2]. The incidence of *BRAF* gene mutation in CMPT was higher than that of *EGFR* and *KRAS* mutations and ALK rearrangement. In the pathological diagnosis, CMPT should be differentiated from isolated peripheral ciliated papilloma, mucinous adenocarcinoma, mucoepidermoid carcinoma, and bronchiolobronchial squamous metaplasia.

Presently, a standard procedure for the treatment of CMPT is absent. Based on the current evidence, wedge resection of the lung with negative pulmonary margin can be used as a primary recommendation. Systematic lymph node dissection or sampling is of little significance. However, in clinical practice, the intraoperative pathological diagnosis of the frozen tumor sample is often difficult for accurate identification of the nature of the lesion, and the CMPT is often misdiagnosed as adenocarcinoma; occasionally, CMPT was found to exist with other lung adenocarcinomas (as in our second patient). Therefore, in this case, it is appropriate for surgeons to treat patients with surgery according to the principles of adenocarcinoma treatment..

Previously, CMPT was considered as a low-grade malignancy; however, all the reported cases including 2 patients at our hospital have not yet been found to recur or metastasize. Therefore, CMPT can be speculated as a benign lesion. Owing to a limited number of cases and short follow-up duration, the long-term physiological behavior of CMPT remains to be observed. Furthermore, as CMPT is very rare and due to the limited knowledge of the pathologist about the disease, CMPT could have been misdiagnosed in the past. Thus, we reviewing the previous pathological sections by the pathologist for a rediagnosis and analyzing these patients for conclusive evidence is imperative.

## Conclusions

CMPT is a nodular tumor commonly seen in the peripheral lung of middle-aged and elderly individuals. The current evidence states that CMPT is a benign tumor; however, some pathological characteristics of CMPT suggested its low-grade malignant potential. Based on the existing evidence, we speculated that sub-lobectomy might be the preferred choice for the treatment of CMPT. Thus, collecting additional clinical data is essential for accurate conclusions.

## Data Availability

All data and material are available by contacting doctorsk@139.com.

## Abbreviations

CMPT: Muconodular papillary tumor
CT: Computed tomography
